# Chronic Infantile Neurological Cutaneous and Articular (CINCA) syndrome: a review

**DOI:** 10.1186/s13023-016-0542-8

**Published:** 2016-12-07

**Authors:** Martina Finetti, Alessia Omenetti, Silvia Federici, Roberta Caorsi, Marco Gattorno

**Affiliations:** 1 UO Pediaria II, G. Gaslini Institute, Genoa, Italy; 2 DINOMGI, University of Genoa, Genoa, Italy

**Keywords:** Autoinflammation, Cryopyrinopathies, Urticarial rash, Aseptic meningitis, Hearing loss, IL-1

## Abstract

**Introduction:**

The Chronic Infantile Neurological Cutaneous and Articular (CINCA, or Neonatal-onset multisystem inflammatory disease NOMID) is a rare autoinflammatory disease identified in 1987 by Prieur et al., typically characterized by the triad of skin rash, arthropathy and central nervous system manifestations. It represents the most severe phenotype of the cryopyrin-associated periodic syndrome (CAPS).

**Clinical description and etiology:**

The syndrome is due to autosomal dominant gain of function mutations in *NLRP3*, which encodes a key component of the innate immunity that regulates the activation and secretion of interleukin (IL)-1β. From the first days of life, patients display an urticarial rash in association with chronic inflammation with a typical facies featured by frontal bossing and saddle back nose. The CNS manifestations include chronic aseptic meningitis leading to brain atrophy, mental delay and sensorineural hearing loss. Chronic polyarthritis and alteration of the growth cartilage also may be present. CINCA/NOMID diagnosis is made clinically, based on the presence of characteristic features. The detection of *NLRP3* mutations is diagnostic in 65–70% of cases. Indeed, up to 40% of affected patients are negative for germline *NLRP3* mutations and several subjects are carriers of somatic mosaicism. Due to the pivotal role of Cryopyrin in the control of Caspase-1 activation and the massive secretion of active IL-1β observed in cryopyrin-mutated individuals, anti-IL1 treatment represents the standard therapy.

**Conclusion:**

Prognosis of CINCA/NOMID syndrome has been changed by the availability of anti-IL1 drugs. Nowadays, the use of anti-IL-1 drugs has sensibly reduced the risk of developing main complications such as severe intellectual disability, hearing-loss and amyloidosis, if treatment is started early on.

## Background

Cryopyrin-associated periodic syndromes (CAPS) are inherited autoinflammatory conditions characterized by chronic systemic inflammation due to an abnormal regulation of the innate immune system. Three diseases of rising severity belong to this group: Familial Cold Autoinflammatory Syndrome (FCAS), Muckle-Wells syndrome (MWS) and Chronic Infantile Neurological Cutaneous and Articular (CINCA, or Neonatal-Onset Multisystem Inflammatory Disease, NOMID), with the latter being the most severe form of CAPS spectrum. Disease onset usually occurs within the first hours/days of life and it is characterized by intermittent fever (that may be of low grade or even absent), urticarial rash and persistent elevation of acute phase reactants. A neurological involvement featured by chronic aseptic meningitis and papilledema is usually present at disease presentation and may lead to brain atrophy, severe intellectual disability and hearing loss. Hypertrophic arthropathy with contractures and bone deformity (frontal bossing, patellar overgrowth) is also typical of this form.

## Disease name/synonyms

Chronic infantile neurological cutaneous and articular (CINCA) syndrome is also called Neonatal onset multisystemic inflammatory disease (NOMID), Infantile-onset multisystem inflammatory disease (IOMID) and Prieur-Griscelli syndrome (OMIM 607115).

## Definition and classification

CINCA/NOMID syndrome is the prototype of an inherited autoinflammatory disorder due to mutation of a gene encoding the NLPR3 (or cryopyrin) protein, which is involved in the activation of the inflammatory response. It belongs to the group of the so-called inflammasome-pathies, together with other conditions associated to mutations of genes that are members of the same protein family (NLRP12, NLRC4, NLRP12).

## Epidemiology

It is estimated that globally the whole spectrum of CAPS has a prevalence of 1–2 cases in every 1 million [1] and 360,000 [2] people in the US and France, respectively. According to a prospective surveillance of children with CAPS performed in Germany during a time period of 3 years by Lainka et al., the incidence of CAPS in Germany corresponds to 2–7 newly diagnosed patients ≤16 years per year [3].

## Clinical description

CINCA/NOMID syndrome represents the most severe phenotype in the context of the clinical spectrum of CAPS (Table 1).

**Table 1:** Cryopyrin associated periodic syndrome (CAPS): main clinical features

Familial cold autoinflammatory syndrome (FCAS)	Muckle–Wells syndrome (MWS)	NOMID/CINCA
• Autosomal dominant	• Autosomal dominant	• Autosomal dominant • Sporadic
• Cold-induced: – Fever – Rash – Conjunctivitis – Arthralgia	• Fever • Urticarial rash • Conjunctivitis • Arthralgia • Sensorineural deafness • AA amyloidosis (in 25% of patients) leading to renal failure	• Fever • Urticarial rash • Conjunctivitis • Visual and intellectual damage • Sensorineural deafness • Progressive chronic meningitis • Destructive arthritis • AA amyloidosis leading to renal failure

In a recent study by Levy et al. [4] the whole spectrum of CAPS in adult and pediatric patients has been described in a large series of 136 patients enrolled in the Eurofever International Registry. The median onset age was 0.8 years (range 0.1–5) while the median age at diagnosis was 15 years (range 5–36) with a mean delay of diagnosis of 14 years. Seventy-eight patients (57%) had a chronic course with symptoms almost daily, whereas fifty-eight (43%) experienced only acute episodes. Fever, skin rash and musculoskeletal involvement were the most prevalent features (observed in 84, 97 and 86% of patients, respectively) and were observed in all the 3 diseases (FCAS, MWS and CINCA/NOMID).

**Fig. 1:**
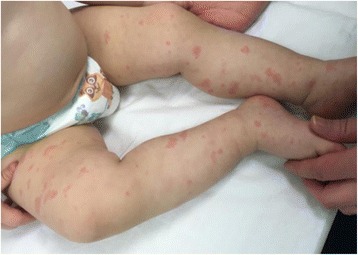
Urticarial rash of CAPS patient

In general, CINCA/NOMID children present during the first days of life with a chronic urticarial rash associated to persistent low-grade fever and sustained elevation of acute phase reactants. The rash is non-pruritic and it changes distribution during the day, without vasculitic alterations (Fig. 1).

**Fig. 2:**
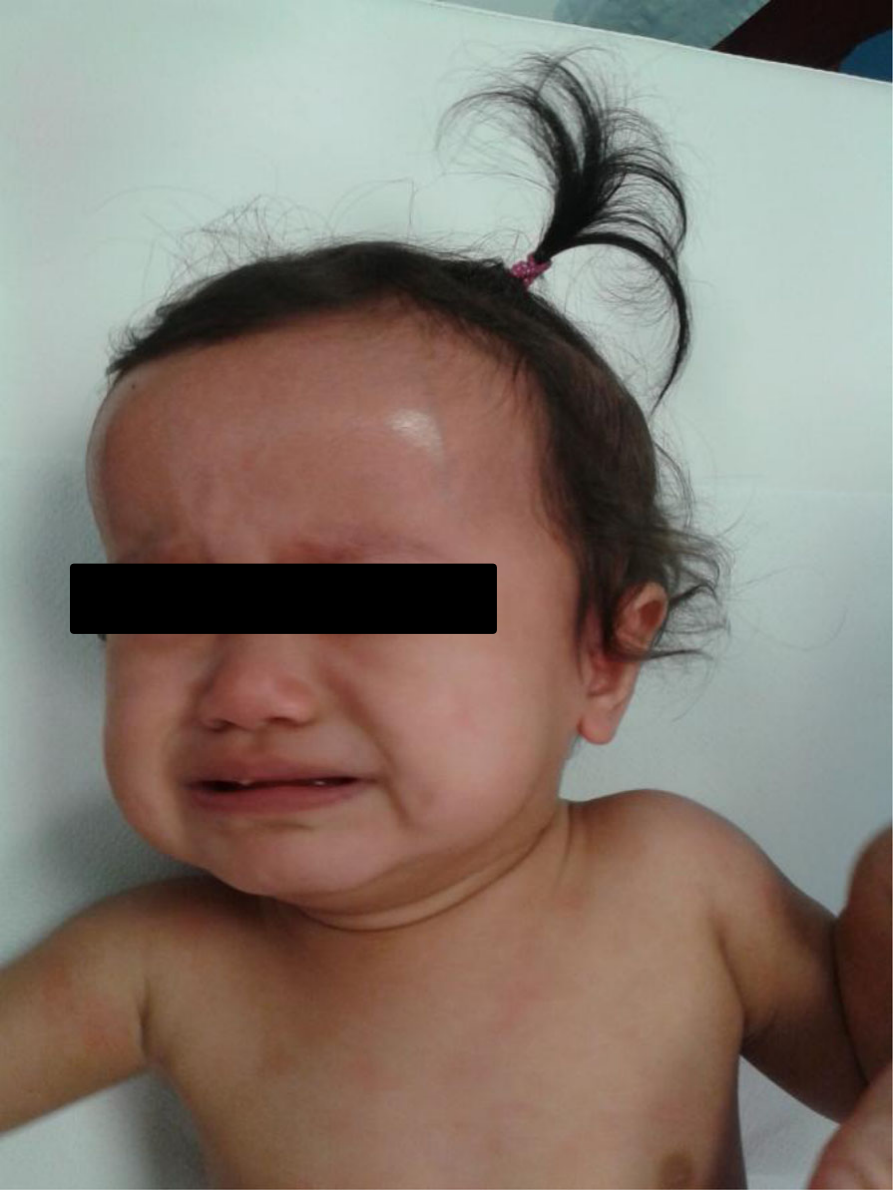
Typical CINCA/NOMID “facies” featured by frontal bossing, large cephalic perimeter and saddle-back nose

Patients display a typical “facies”, featured by frontal bossing, large cephalic perimeter and saddle-back nose (Fig. 2).

**Fig. 3:**
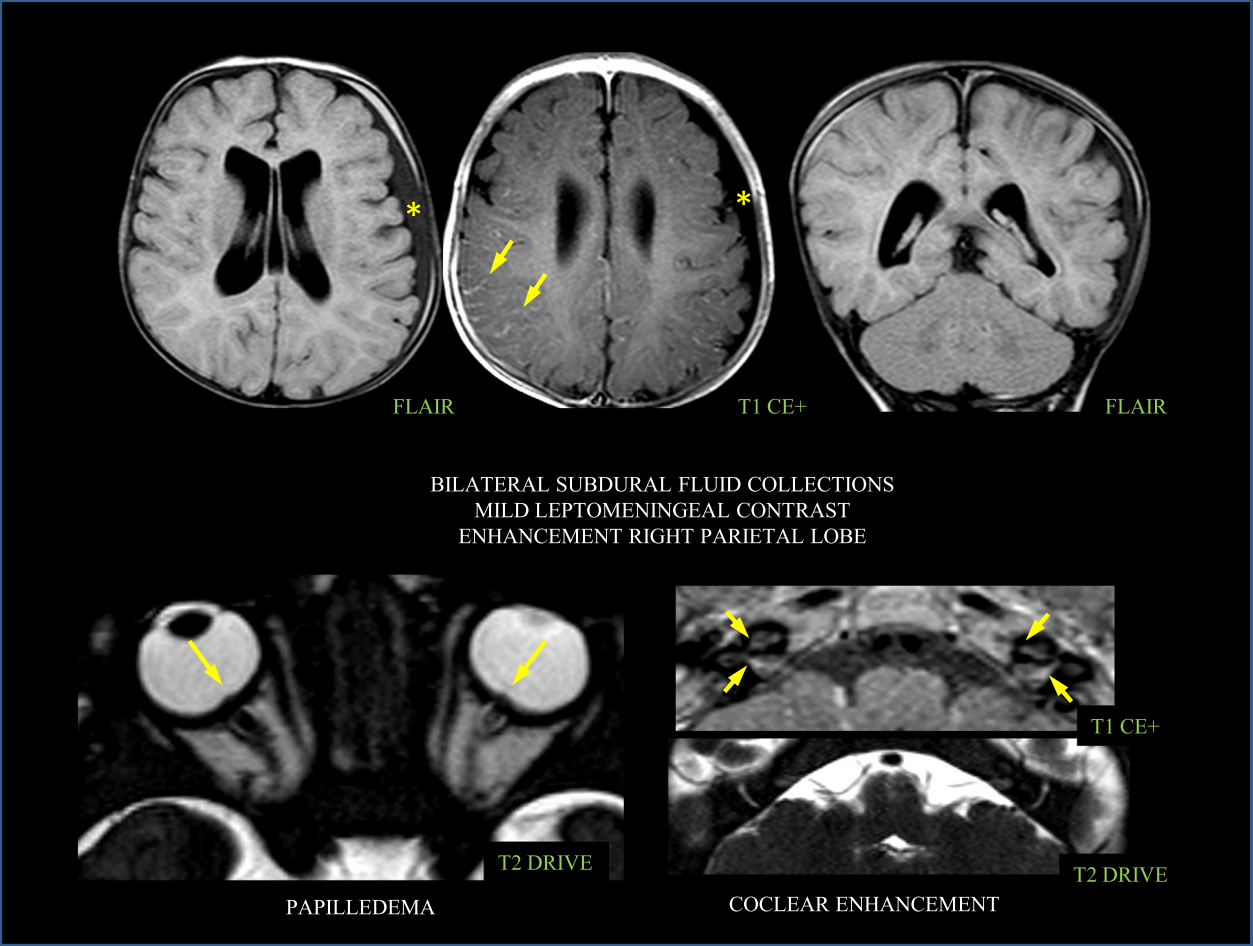
MR features of CINCA/NOMID brain (subdural fluid collections, leptomeningeal contrast, papilledema, coclear enhancement)

If untreated, these patients develop permanent CNS damages as consequence of chronic inflammation [5]. Chronic aseptic meningitis may cause intracranial pressure to increase, resulting in hydrocephalus, brain atrophy and chronic papilledema (Fig. 3). Neurological symptoms typical of CINCA/NOMID are characterized by chronic irritability, intellectual disability, headache, early morning nausea, vomiting and, rarely, seizures. In the Eurovefer registry the most frequent severe neurological manifestation was chronic meningitis, that was observed in 26% of patient. Other major neurological features (seizures, hydrocephalus, mental delay) were reported in 12% of the whole CAPS population.

The most frequent inflammatory eye manifestation is conjunctivitis. Persistent papilledema is rather common and it may cause optic nerve atrophy with progressive vision loss if left untreated [6, 7]. Anterior uveitis can contribute to the progressive vision loss. In the study of Dollfus et al. [8] the ocular manifestations in CINCA/NOMID patients (*n* = 31) were described with a mean follow up of 11.5 years (2 to 32 years). The first ocular sign occurred at the mean age of 4.5 years (1–9 years), and it was represented by red eyes (*n* = 9), decreased vision (*n* = 7), strabismus (*n* = 4), nystagmus (*n* = 2) and photophobia (*n* = 2). During follow-up, 8 patients complained visual loss, all of them had signs of optic nerve lesion (i.e. optic disc edema in 2 patients and optic atrophy in 6 subjects), while 6 had also corneal involvement. In most patients, anterior ocular inflammation was featured by a mild to moderate anterior uveitis (9 out of 16 patients), while posterior inflammation seemed to occur less frequently. Optic disc abnormalities occurred in 26 patients (84%) and included optic disc edema and, less frequently, pseudopapilledema and optic atrophy. Anterior segment manifestations were observed in 13 patients (42%). Disease-related visual acuity loss in at least 1 eye was reported in 8 patients (26%). Posterior synechia, glaucoma, and white iritis were not observed in any patient.

Persistent cochlear inflammation may lead to sensorineural hearing loss usually occurs within the first years of life in a relevant percentage of untreated patients (42% of CAPS patients enrolled in Eurofever Registry, Levy et al.). In a prospective study performed in 2013 by Neda Ahmadi et al. [9], data regarding clinical aspects, audiologic phenotype and fluid attenuation inversion recovery MRI (FLAIR-MRI) were collected in CAPS patients (*n* = 57) including 31 CINCA/NOMID subjects. Complete audiological data were obtained in 70% of ears and displayed conductive, mixed and sensorineural hearing loss in 11, 13 and 61% of CINCA/NOMID ears, respectively. The latter was worse in the higher frequencies. Cochlear enhancement on FLAIR-MRI sequences was observed mainly in CINCA/NOMID (*n* = 26) and it appeared to be an accurate predictor of cochlear hearing loss.

Approximately 60% of CINCA/NOMID patients have prominent arthropathy, most frequently involving large joints as knees [10]. It usually begins during childhood and causes deformities that persist in adult age, resulting in early degenerative arthropathy and joint contractures. Histological analysis of growth cartilage displays a complete disorganization and irregular metachromasia of cartilage substance, but no inflammatory cell infiltrates [11]. In a 2007 study by Hill et al., radiographs were performed in a total of 20 CINCA/NOMID patients and bone abnormalities were detected in 11 out of 20 knees of patients. These findings included enlargement and deformities of femora and patellae in all the patients, without evidence of synovitis. The mechanism leading to articular damage is likely represented by an abnormal endochondral bone formation. Patellar premature ossification and overgrowth are typical, although rare, findings in CINCA/NOMID [7]. A chronic polyarthritis may also be present, sometimes leading to bone erosions. The multisystemic involvement characteristic of CINCA/NOMID syndrome has a severe impact on the quality of patient life [12].

## Aetiology-genetics

As aforementioned, CINCA/NOMID represents the most severe phenotype of CAPS, monogenic diseases due to autosomal dominant inheritance of mutations in the *NLRP3* gene (formerly known as cold-induced autoinflammatory syndrome 1, *CIAS1*), which encodes the NLRP3 protein also termed cryopyrin. *NLRP3* variants causing CAPS are missense mutations resulting in a *gain of function* that, thus, enhance NLRP3 activity. To date, approximately 170 *NLRP3* variants have been described and associated to either CINCA/NOMID or to milder phenotypes of the NLRP3-driven clinical spectrum including FCAS and MWS (http://fmf.igh.cnrs.fr/ISSAID/infevers/) [9]. Almost all the *NLRP3* mutations so far identified have been observed within the exon 3, which is responsible for encoding the NACHT domain, crucial for cryopyrin oligomerization. However, although patients with FCAS and MWS tend to show familial inheritance patterns, CINCA/NOMID syndrome usually occurs *de novo*, and approximately 50–65% the patients with a CINCA/NOMID phenotype lack detectable mutations in the *NLRP3* coding region [13–16]. It has been suggested that different genes or a modifier gene could be involved in these latter cases, although they do not differ clinically from those carrying *NLRP3* mutations. In the past few years somatic *NLRP3* mosaicism rather than heterozygous germ-line mutation has been detected in up to 69.2% [17] of patients presenting symptoms typical of CINCA/NOMID [17–22]. Thus, for a majority of genetically negative CINCA/NOMID patients, the disease onset may be caused by low-level mosaicism in the absence of detectable *NLRP3* gene mutations by ordinary genomic sequencing [18–20]. *NLRP3* mosaicism is thus an established major cause of CINCA/NOMID [17]. However, only recently, insights regarding the relevance of this mechanism were provided in other CAPS phenotypes [23, 24]. Namely, a variable degree (5.5–34.9%) of somatic *NLRP3* mosaicism vertical transmission was detected in patients with a MWS phenotype, pointing at somatic *NLRP3* mosaicism as shared genetic mechanism in the whole CAPS spectrum, not restricted to CINCA/NOMID clinical picture [23, 24].

## Aetiology- pathophysiology

**Fig. 4:**
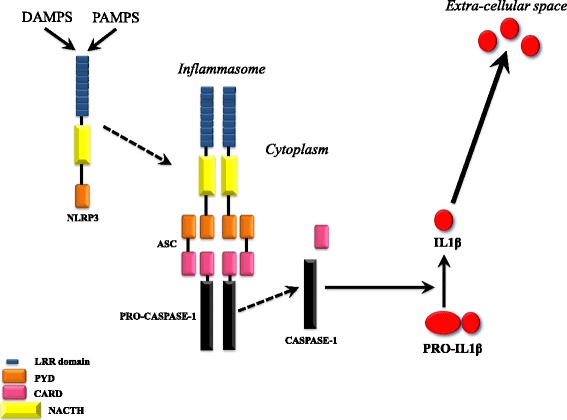
Schematic illustration of the NLRP3 inflammasome activation

The main functional effect of genetic background causing CAPS is the abnormal activation of innate immunity cells by endogenous and exogenous stimuli. CAPS may be considered the prototype of monogenic autoinflammatory diseases that, compared to autoimmune conditions, are featured by an apparent secondary role of the adaptive branch of the immune response, as suggested by the lack of self-reactive T cells and/or circulating autoantibodies and by the absence of a clear class II MHC association [25]. NLRP3 actually belongs to Nucleotide-binding domain and Leucine-rich repeat containing Receptor (NLRs) family (also known as Nucleotide-binding Oligomerization Domain (NOD)–Like Receptors), which act as cytosolic pattern-recognition receptors (PRRs) [26]. As a NLRs member, NLRP3 physiologically functions as danger sensor and undergoes activation following several insults, including both exogenous Pathogen-Associated Molecular Patterns (PAMPs) and the endogenous Damage-Associated Molecular Patterns (DAMPs) [27, 28]. Once triggered, NLRP3 interacts with other intracellular proteins and assemblies into a multiprotein complex called Inflammasome, a key player in IL-1β pathway activation. Unlike most cytokines, indeed, the pro-inflammatory IL-1β lacks a secretory signal peptide and it is released by monocytes through a non canonical pathway including two steps [29–31]. (Fig. 4) First, the 33-kD IL-1β precursor pro-IL-1β is induced by bacterial products (e.g. lipopolysaccharide, LPS) and accumulates in the cytosol. Secondarily, when a second hit triggers NLRP3, the inactive pro-IL-1β is converted into its mature 17-kD form [32]. Following activation, NLRP3 oligomerizes and binds the adaptor Apoptosis-associated Speck-like protein containing a CARD (ASC) via its Pyrin (PYD) domains. NLRP3-ASC complex then directly activates pro-caspase1 into the active proteolytic enzyme caspase1, which, in turn, cleaves the IL-1β and IL-18 precursors into their bioactive forms [32]. This tight and complex control of IL-1β processing and secretion is linked to the powerful properties of this proinflammatory cytokine and it is necessary in order to avoid adverse effects related to its excessive release [33, 34]. The unrestrained IL-1β secretion occurring in CAPS patients further proves this concept. CAPS, indeed, may be actually considered intrinsic cryopyrinopathies in which *gain of function* mutations primarily affecting NLRP3 cause cells to display a constitutively turned-on inflammasome [35]. This condition releases inflammasome from the need of a normally required second signal (i.e. ATP), leading to caspase-1-driven IL-1β over-production [36]. Several data derived from either in vitro, in vivo and *ex vivo* studies unveiled that NLRP3 mutations and redox alterations work together in determining CAPS IL-1β pattern of secretion. Indeed, factors inducing cellular stress may give rise to persistent activation of innate immunity. In the presence of overly active danger sensor such as NLRP3, it is conceivable that IL-1-mediated signaling cascade may be enhanced. Reactive oxygen species (ROS)-associated genes represent, indeed, one of most differentially expressed set in severe CAPS [37]. For years, oxidative stress has been investigated as potential key player in the activation of NLRP3-inflammasome [34, 38]. NLRP3-mutated CAPS monocytes basally display elevated levels of ROS and fragmented mitochondria, and these stress marks are dramatically worsened by TLR stimulation [39]. CAPS resting monocytes also exhibit higher levels of antioxidants, and redox response to PAMPs is also altered, with faster upregulation of the antioxidant machinery which rapidly undergoes exhaustion [40]. As result, CAPS patients are uniquely featured by a dramatically anticipated IL1β secretion [40]. As aforementioned, once elicited, IL-1β pathway disruption occurring in cryopyrinopathies triggers a cascade of complex cellular events leading to aberrant homeostatic tissue responses. In conclusion, the research field exploring CINCA/NOMID pathogenesis is continuously in progress. A recent newly identified mechanism based on NLRP3 inflammasome acting also as an extracellular oligomeric complex thus causing persistent inflammatory response further expands the growing complexity underlying CINCA/NOMID pathogenesis and open the field to a wide spectrum of cross-talking signaling potentially involved [41].

## Diagnosis and diagnostic methods

The characteristic clinical picture, starting with a persistent urticarial rash associated to systemic inflammation signs during the first days or week of life usually leads to the suspicion of a diagnosis CINCA/NOMID syndrome [42]. The persistence of the symptoms despite the antibiotic and/or antiviral treatment and the partial control of the symptoms achieved by steroids have to address the suspicion through a cryopyrin associated periodic syndrome. Even if laboratory alterations are not specific, CINCA/NOMID patients may display persistent elevation of acute phase reactants, leukocytosis and chronic anemia. Recently, evidence-based clinical criteria for CAPS and other inherited periodic fevers have been developed, on the basis of the multivariate analysis performed on a large number of patients enrolled in the Eurofever international registry [43]. Even if these criteria were developed in patients presenting a recurrent disease course, 100% of the CINCA/NOMID patients enrolled in the Eurofever registry were correctly identified. New specific criteria for CAPS, including the most severe CINCA/NOMID phenotype are currently under development (Kummerle et al., in preparation). In order to confirm the diagnosis the molecular analysis of *NLRP3* gene is surely required. Due to the severe phenotype, usually *de-novo* mutations are detected. As already stated, up to 35–40% of patients with a clear CINCA/NOMID phenotype turn out to be negative for germ-line mutations of *NLRP3* [17]. Almost 70% of these patients are, instead, carriers of a somatic mosaicism [44], that can involve even a very low percentage of the cells of myeloid lineage [17]. There is a general consensus among experts that the clinical picture of CINCA/NOMID is sufficient to point out the diagnosis even in the absence of a positive genetic test [45].

## Differential diagnosis

The disease onset of CINCA/NOMID syndrome usually occurs early on, often within the first months of life. Therefore, in the beginning, an infectious disease is often suspected. A diagnostic algorithm in children with fever and skin rash has been recently developed in order to differentiate CINCA/NOMID from the other cryopyrinopathies (FCAS and MWS) and autoinflammatory diseases (i.e. CANDLE syndrome and systemic onset juvenile idiopathic arthritis) characterized by a similar clinical picture [42]. Patients with a severe form of mevalonate kinase deficiency (MKD) may present a very early onset with a chronic and subchronic disease course. An urticarial skin rash can also be present, even if without the daily occurrence observed in CAPS. TNF-receptor associated periodic fever (TRAPS) usually present with long lasting fever episodes followed by clinical remission. An urticarial skin rash has been observed, at least occasionally, in 25% of TRAPS patients enrolled in the Eurofever registry [46]. However, the presence of other more typical manifestations (erythematous migratory rash, fasciitis, periobrital oedema, abdominal and chest pain) help in the differentiation with CAPS. Due to the lack of urticarial rash, other forms of periodic fever, such as familiar Mediterranean fever (FMF) and PFAPA (periodic fever with adenitis pharingitis and aphtosis) syndrome do not usually enter in the differential diagnosis with CAPS.

## Genetic counseling

Patients affected by CINCA/NOMID syndrome and carrying germinal mutation in *NLRP3* gene have the chance of 50% to transmit the disease to each child, since the modality of transmission is autosomal dominant. In case of somatic mosaicism this risk depends on the possible presence of somatic mutations in reproductive organs in the parents [23].

## Antenatal diagnosis

In presence of an affected parent the search for the *NLRP3* mutations can be performed through prenatal diagnosis techniques (chorionic villus sampling or amniocentesis). The availability of anti-IL-1 treatment has dramatically changed the long-term prognosis and the quality of life of CAPS patients with a mild and intermediate phenotypes that are usually transmitted with the typical autosomal pattern. Conversely, the presence of a severe neurological involvement in patients with CINCA/NOMID phenotype might be associated to the possibility to develop various degree of neurological complications and hearing loss, even with an adequate and timely treatment (see below). As stated above this latter condition is usually associated to the presence of de-novo mutations or somatic mosaicisms. This should be carefully considered in the process of genetic counseling, concerning possible prenatal and/or pre-implantation genetic diagnosis.

## Management including treatment

The discovery of the mechanism of action of cryopyrin in the control of caspase-1 activation and the aberrant secretion of IL-1β observed in patients with this disease suggested that IL-1 blockage might be an effective treatment (Table 2).

**Table 2:** Results of the main studies analyzing the use of antiIL1 drugs in CINCA syndrome

Study	Type of study	Type of drug	Number of patients enrolled	Study duration (months)	Number of patients who reached primary endpoint	Number of patient with central nervous system remission^a^
Goldbach-Mansky et al. 2006 [7]	Open-label	anakinra	18 CINCA	6	18/18	In 12 patients intracranial pressure, protein levels and white cells count decreased significantly
Lepore et al. 2010 [12]	Open-label	anakinra	14 CAPS: -10 CINCA - 4 MWS	36	14/14	Not evaluated
Sibley et al. 2012 [5]	Open-label	anakinra	26 CINCA	60	26/26	Significant decrease of CSF leukocyte count at 36 and 60 months (*p* = 0.0026 and 0.0076)
Hoffman et al. 2012 [58]	Open-label	rilonacept	101 CAPS (FCAS or MWS)	18	Mean key symptom score at week 72 reduced from 2.6 to 0	Not evaluated
Caorsi et al. 2013 [53]	Open-label	canakinumab	13 CAPS: - 7 CINCA - 4 MWS - 2 overlap CINCA/MWS	12	Complete response^b^: Baseline: 5/13 Last Follow-up: 8/13	Not evaluated
Sibley et al. 2015 [55]	Open-label	canakinumab	6 CINCA	24	Full remission^c^ at month 6: 0/6 Inflammatory remission^d^ at month 6: 4/6	6 months: 0/6

^a^CNS involvement: abnormal CSF leukocyte count; ^b^complete response: absent or minimal disease activity at the global assessment with acute phase reactants within the normal range; ^c^Full remission: remission of patient-reported clinical components and measures of systemic inflammation and CNS inflammation; ^d^Inflammatory remission: CRP ≤10 mg/L and global diary score remission

Initially, isolated case reports and small studies showed the dramatic effects of IL-1 receptor antagonist (anakinra) in the control of rash and other systemic manifestations in MWS [45], FCAS [47] and CINCA/NOMID [7, 36, 48] patients. The long-term efficacy and safety of anakinra in CINCA/NOMID were described two distinct studies conducted in Italy and France, respectively [12, 49]. Namely, data derived from both analyses indicated that anakinra regimen was safe and effective on the long-term, and that it was associated with a dramatic amelioration of the quality of life [12]. Sibley et al. published an open-label, long-term follow-up study on a cohort of 26 CINCA/NOMID patients treated with anakinra 1–5 mg/kg/day for at least 36 months [5]. In particular, the study aimed at evaluating the efficacy and safety of anti-IL-1 therapy in controlling systemic and organ-specific inflammation and in preventing the progression of organ damage. Sustained improvements in diary scores, parent’s/patient’s and physician’s global scores of disease activity, parent’s/patient’s pain scores, and inflammatory markers were observed during all the period of the study. Despite a general good control of clinical manifestations (including hearing loss, ocular manifestations and headache) and laboratory parameters, few patients displayed a persistent, although mild, inflammation of CNS, and treatment with anti-IL-1 did not prevent the progression of the bone involvement. Overall, this study provided evidence of sustained efficacy of anti-IL-1 in CINCA/NOMID without occurrence of significant adverse effects for up to 5 years. Anakinra was approved for the CINCA/NOMID form of CAPS in children older than 8 months in the US in early 2013 and for all forms of CAPS (FCAS, Muckle-Wells and CINCA/NOMID) in the EU in November 2013. The same good results have been also achieved with other newer anti-IL-1 including rilonacept (a fully human dimeric fusion protein that incorporates the extra-cellular domain of both IL-1 receptor components) and canakinumab (a fully human anti-interleukin-1β monoclonal antibody that selectively blocks IL-1β). Since the efficacy of rilonacept has been demonstrated only in patients with the milder phenotype [50], this drug is approved for FCAS or MWS patients older than 11 years in US. Canakinumab is used subcutaneously at a starting dose of 150 mg (or 2 mg/kg) every 8 weeks [51]. However, as described in the following paragraphs, patients with CINCA/NOMID syndrome often require a higher dose (4 mg/kg) and frequency of administration [52, 53] Canakinumab is now approved in Europe and US for the use in all subtypes of CAPS in patients older than 2 years. The percentage of complete response to these drugs is generally quite high. However, a partial response, mainly related to a persistent elevation of acute phase reactants despite a satisfactory control of the clinical manifestations has been reported either with the use of Anakinra and Canakinumab in the Eurofever registry [54]. In Kuemmerle-Deschner et al. 2011 study [52], an open-label, multicentre, phase III study evaluating the safety and efficacy of canakinumab in the largest cohort of CAPS subjects, 32 CINCA/NOMID patients received subcutaneous canakinumab (i.e. 150 mg or 2 mg/kg ≤40 kg every 8 weeks) for up to 2 years, and changes from baseline in clinically significant abnormality of neurological, audiogram and ophthalmological assessments were evaluated. Regarding neurological involvement, 4 CINCA/NOMID patients had abnormal findings at baseline (i.e. bilateral hearing loss, polyneuropathy, bilateral carpal tunnel syndrome, language-based learning disability, chronic headache). The successive neurological assessments performed in the next 2 years during canakinumab therapy showed normalization of these findings in one CINCA/NOMID patient. Concerning audiological impairment, in 4 CINCA/NOMID patients (aged 3–24 years) the abnormal audiogram findings remained unchanged despite the 2 years treatment with canakinumab. Finally, the resolution of macular oedema was observed for 1 eye of a CINCA/NOMID patient and clinical improvement in blepheratis was recorded in another CINCA/NOMID subject. Notably, during this study almost 50% of CINCA/NOMID patients required an adjustment of the dose and/or frequency of drug administration [52]. In a subsequent open study conducted in Italy in patients previously enrolled in the aforementioned trial, the median dose of canakinumab in CINCA/NOMID patients after 12 months was 4 mg/kg with a mean frequency of administration of 5 weeks [53], suggesting that the initial dose of canakinumab in CINCA/NOMID patients should be higher than 2 mg/kg every 8 weeks, which, instead, represents the staring dose in the milder forms of CAPS spectrum such as FCAS and MWS. Due to the extreme rarity of the disease, few data are available on treatment of most severe CINCA/NOMID patients during the first year of life. Unfortunately, this period is absolutely critical because most of the cerebral damage secondary to the persistent inflammation occurs right in the early phases of the mental development. Thus, a crucial point in CINCA/NOMID patients with severe phenotype is the therapeutic management during the first year of life. While, in fact, it has been demonstrated in non-human primates that the diffusion of anakinra in the CNS is proportional to the systemic dose, there is no clear evidence regarding whether, and in which proportion, the new anti-IL-1 drugs may be actually capable of passing through the blood–brain barrier therefore allowing to control the disease activity in the CNS in these patients with more severe CINCA/NOMID phenotype. Six patients were enrolled and treated with canakinumab after anakinra withdrawal in a recent open-label study [55]. In this study, all 6 patients required a dose escalation (i.e. from 150 mg or 2 mg/kg in patients <40 Kg to 600 mg or 8 mg/kg in patients <40 Kg every 4 weeks). Treatment with canakinumab improved symptoms and serum inflammatory signs, although low-grade CNS leukocytosis persisted in 4 patients and headaches was still affecting 1 patient despite ongoing treatment. In patients with CINCA/NOMID that progressed into intellectual disability, speech and psychomotor therapy were often required. Hearing loss often led to the need of supporting hearing devices or cochlear implants. Thus, the daily clinical management of patients with CINCA/NOMID is extremely complex and only recently, evidence-based recommendations have been developed with particular attention to the management of all form of CAPS spectrum and other autoinflammatory diseases. According to these recommendations, use of anti-IL-1 is indicated for whole spectrum of CAPS, at any age (level of evidence 1B-2A) and long-term IL-1 inhibition should be started as early as possible in patient with active disease to prevent organ damage [56]. There is general consensus on the opportunity to ask for a specific anti-IL-1 treatment also in those patients with a clinical picture highly consistent with a CAPS phenotype that turned out to be negative for germ-line mutations [45].

## Prognosis

Intellectual disability is often enlightened as the main relevant prognostic factor and it develops in the first years of life. Its severity is proportional to the delay of the diagnosis and of the beginning of appropriate anti-IL-1 treatment. Prognosis of CINCA/NOMID syndrome has been dramatically improved by the availability of anti-IL-1 drugs. In the past, the life prognosis was significantly affected by several complications, including the development in the adult age of renal amyloidosis, responsible for the occurrence of chronic renal failure. Even if no direct evidences are available so far, it is conceivable that the dramatic control of the systemic inflammation using anti-IL-1 will sensibly reduce the risk of the occurrence of this complication. Lane et al. in 2013 described 4 CAPS patients with amyloidosis treated with anti-IL-1 agents. Of the 4 patients, 1 had received a renal transplant prior to initiation of this treatment; all the remaining 3 patients have a resolution of proteinuria over the follow-up period. However, 3 of the 4 patients had a stable chronic kidney disease and in 1 patient disease progressed from stage 1 to stage 2 over the 11-years follow-up period. These findings suggest that even in patients with established amyloidosis, effective treatment of the underlying syndrome can lead to improved renal function and regression of amyloid, as long as renal impairment is not too advanced at time of diagnosis [57]. It may be reasonable that patients that have been treated since the first months of life will present a consistent reduction of long-term complication. However the progression of hearing loss has been described even in children that received a prompt diagnosis and treatment. In this line, more efforts are needed to provide evidence on the best possible treatment, dosage and frequency of administration of anti-IL-1 treatment in order to completely prevent the occurrence of these complications.

## Unresolved questions

CINCA/NOMID syndrome is a relatively new disease and the research exploring the underlying pathogenetic mechanisms is constantly growing. Moreover, while the clinical outcome is known for those patients who did not receive treatment with anti-IL-1, the outcome through adulthood of the patients who received anti-IL-1 treatment in the infancy is still unknown. Regarding the new anti-IL-1 drugs, the percentage of these drugs reaching the CNS and therefore the possibility to prevent the development of intellectual disability and brain atrophy, above all in young children, it is unclear.

## Conclusion

CINCA/NOMID syndrome is a rare autoinflammatory syndrome. Recently, the pivotal role of IL-1 in the disease pathogenesis allowed to develop standard therapy with anti-IL-1 agents that have dramatically changed the prognosis during last years.

## Abbreviation

ASC: Apoptosis-associated speck-like protein containing C-terminal caspase recruitment domain
CANDLE: Chronic Atypical Neutrophilic Dermatosis with Lipodystrophy and Elevated temperature
CAPS: Cryopyrin-associated periodic syndromes
CARD: Caspase activation and recruitment domains
CINCA: Chronic infantile neurological cutaneous and articular syndrome
CNS: Central nervous system
DAMPs: Damage-Associated Molecular Patterns
FCAS: Familial cold autoinflammatory syndrome
IL-1: Interleukin1
LPS: Lipopolysaccharide
MHC: Major Histocompatibility Complex
MRI: Magnetic Resonance Imaging
MWS: Muckle-Wells syndrome
NLRs: Leucine-rich repeat containing Receptor
NOD: Nucleotide-binding Oligomerization Domain
NOMID: Neonatal-onset multisystem inflammatory disease
PAMPs: Pathogen-Associated Molecular Patterns
PRRs: Pattern-recognition receptors
PYD: Pyrin domain
ROS: Reactive oxygen species
SNHL: Sensorineural hearing loss
TLR: Toll like receptor
